# An Estimate of the Incidence and Prevalence of Stroke in Africa: A Systematic Review and Meta-Analysis

**DOI:** 10.1371/journal.pone.0100724

**Published:** 2014-06-26

**Authors:** Davies Adeloye

**Affiliations:** Centre for Population Health Sciences, University of Edinburgh Medical School, Edinburgh, Midlothian, United Kingdom; Innsbruck Medical University, Austria

## Abstract

**Background:**

Stroke is increasingly becoming a challenging public health issue in Africa, and the non-availability of data has limited research output and consequently the response to this burden. This study aimed to estimate the incidence and prevalence of stroke in Africa in 2009 towards improved policy response and management of the disease in the region.

**Methods:**

A systematic search of Medline, EMBASE and Global Health for original population-based or hospital-based studies on stroke was conducted. A random effect meta-analysis was conducted on crude stroke incidence and prevalence rates, and a meta-regression-like epidemiological model was applied on all data points. The fitted curve generated from the model was used to estimate incident cases of stroke and number of stroke survivors in Africa at midpoints of the United Nation population 5-year age groups for the year 2009.

**Results:**

The literature search yielded a total of 1227 studies. 19 studies from 10 African countries were selected. 483 thousand new stroke cases among people aged 15 years or more were estimated in Africa in 2009, equivalent to 81.2 (13.2–94.9)/100,000 person years. A total of 1.89 million stroke survivors among people aged 15 years or more were estimated in Africa in 2009, with a prevalence of 317.3 (314.0–748.2)/100000 population. Comparable figures for the year 2013 based on the same rates would amount to 535 thousand (87.0–625.3) new stroke cases and 2.09 million (2.06–4.93) stroke survivors, suggesting an increase of 10.8% and 9.6% of incident stroke cases and stroke survivors respectively, attributable to population growth and ageing between 2009 and 2013.

**Conclusion:**

The findings of this review suggest the burden of stroke in Africa is high and still increasing. There is need for more research on stroke and other vascular risk factors towards instituting appropriate policy, and effective preventive and management measures.

## Introduction

The burden of stroke is increasing in many low- and middle-income countries (LMIC) [1], and due to high fatality rates and overwhelming resource incurred by the health systems, stroke and many non-communicable diseases (NCDs) are now targeted public health priorities in these regions [2], [3]. Globally, about 16 million new cases of stroke and 62 million stroke survivors were estimated in 2005, with deaths from stroke accounting for 9.7% of all global deaths, and this is expected to increase to over 23 million new stroke cases and 7.8 million stroke deaths by 2030 in the absence of significant global public health response [4], [5].

It has been estimated that LMIC account for over 87% disability adjusted life years (DALYs) from stroke, which is about seven times the DALYs lost in high-income countries (HIC) [6]. Africa is particularly worst hit, owing to population growth, unchecked industrialization and increased consumption of western diets, leading to a rise in many modifiable vascular disease risk factors including smoking, harmful use of alcohol, physical inactivity and unhealthy diets, and invariably resulting in increased prevalence of hypertension, diabetes and obesity [7], [8]. In 2000, two African countries, although recorded low stroke prevalences, had remarkably high stroke incidence rates [9]. According to GBD 2002 estimates, three African countries (Angola, Liberia and Sierra Leone) recorded the highest stroke mortalities and DALYs worldwide [5], [10]. Between 2002 and 2004, estimates further revealed an increasing prevalence with 8% of new stroke cases and 5% of stroke survivors occurring in Africa [5], [11]. Even with this increasing burden, the public health response, accesses to health services and treatment options in many African countries have been poor [7], [12]. Specifically, the lack of functional stroke units, neurologists, health workers, cranial computed tomography (CT) scans, magnetic resonance imaging (MRI) machines and echo-doppler machines, among many others, has negatively affected stroke outcomes [2], [12]. Moreover, the high cost of medical care in a relatively low-income African society could have resulted in high stroke fatalities, as some studies have indicated that stroke prevalence and deaths in Africa increased due to an overtly poor socioeconomic status [6]. For example, a recent study revealed the incidence of stroke in HIC decreased by over 40% between 1970 and 2008, but with actual number of stroke cases increasing due to ageing of the population [13], while in Africa and many LMIC, stroke incidence rose by over 100% over the same period [13]. Furthermore, due to the high proportion of undiagnosed hypertension in Africa especially among the younger population [14], stroke incidence has also been reported to be more severe and higher among the active and productive population age groups [15].

Meanwhile, the World Health Organization (WHO) technically supported her member countries with methods for improved data collation and registration of hospital stroke cases [16]. Notwithstanding, another set-back in the response to the management of stroke in Africa is the lack of data and low research output [7], [14]. Stroke case ascertainment and survey methodologies have not, in most cases, complied with international protocols [7]. Published research studies are characterized by poorly organized community-based studies, difficulties in making retrospective diagnosis, and overlapping cases of first and recurrent strokes [7], [17]. The few studies on stroke, therefore, could have been marked by under-estimation of the stroke burden in Africa. In view of this high burden of stroke, its public health importance, and the relatively low research output in Africa, this study aimed to estimate the incidence and prevalence rates of stroke in Africa in order to attempt to quantify the burden and inform decision regarding policy responses and health system interventions across many countries in the region.

## Methods

### Search strategy and selection criteria

After identification of relevant Medical Subject Headings (MESH) and keywords, a final search strategy was developed. Searches were conducted in three main databases: Medline, EMBASE and Global Health. The search date was set from January 1970 to December 2013. An additional search was conducted on Google Scholar and reference lists of relevant studies to identify publications that could have been omitted in the database searches. The search terms employed on Medline are shown in **Table 1**, while those employed on other databases are shown in **Table S1** and **Table S2** in **File S1**.

**Table 1:** Search terms (Medline).

#	Searches
**1**	africa/or africa, northern/or algeria/or egypt/or libya/or morocco/or africa, central/or cameroon/or central african republic/or chad/or congo/or "democratic republic of the congo"/or equatorial guinea/or gabon/or africa, eastern/or burundi/or djibouti/or eritrea/or ethiopia/or kenya/or rwanda/or somalia/or sudan/or tanzania/or uganda/or africa, southern/or angola/or botswana/or lesotho/or malawi/or mozambique/or namibia/or south africa/or swaziland/or zambia/or zimbabwe/or africa, western/or benin/or burkina faso/or cape verde/or cote d'ivoire/or gambia/or ghana/or guinea/or guinea-bissau/or liberia/or mali/or mauritania/or niger/or nigeria/or senegal/or sierra leone/or togo/
**2**	exp vital statistics/or exp incidence/
**3**	(incidence* or prevalence* or morbidity or mortality).tw.
**4**	(disease adj3 burden).tw.
**5**	exp "cost of illness"/
**6**	exp quality-adjusted life years/
**7**	QALY.tw.
**8**	Disability adjusted life years.mp.
**9**	(initial adj2 burden).tw.
**10**	exp risk factors/
**11**	2 or 3 or 4 or 5 or 6 or 7 or 8 or 9 or 10
**12**	stroke/or brain infarction/or brain stem infarctions/or cerebral infarction/or stroke, lacunar/
**13**	cerebrovascular accident.mp.
**14**	cerebrovascular disease.mp.
**15**	CVA.mp.
**16**	12 or 13 or 14 or 15
**17**	1 and 11 and 16

Studies included for further screening were mainly population/community- and hospital-based studies on stroke in Africa, conducted on or after 1970 and providing numerical estimates on the incidence and/or prevalence of stroke in the region. African countries were as defined by the World Bank list of economies (October 2013) [18]. Studies conducted before 1970, without numerical estimates, on non-human subjects, and that were mainly reviews were excluded. Studies with well-defined stroke diagnostic criteria and survey protocols were further retained. Due to the paucity of data, varying sources of information including demographic health surveys, community-based door-to-door surveys, hospital records and outpatient clinics were allowed. However, the final stroke case ascertainment complied with the standard WHO definition, defined as “rapidly developing clinical signs of focal (or global) disturbance of cerebral function lasting longer than 24 hour, unless interrupted by death, with no apparent cause other than that of vascular origin” [19], [20]. According to experts, new cases of stroke were defined as number of people presenting with first ever stroke in a given period, while stroke survivors were the total number of people who have had stroke or living with its sequelae at a given time [20], [21]

### Data extraction and statistical analysis

An independent parallel search and double extraction was conducted and all extracted data was stored in a Microsoft Excel file format. Data were abstracted systematically on study location, study period, mean age or age range, person years or sample size, incident cases of stroke or number of stroke survivors, and their respective age- and sex-specific incidence or prevalence rates. These were sorted into population-based or hospital-based data separately for analysis. For studies conducted on the same study site, population or cohort, the first chronologically published study was selected, and all additional data from other studies were compared for consistency and included in the selected paper.

From reported overall crude incidence or prevalence of stroke in a given cohort, a random effect meta-analysis was conducted with pooled effect of stroke expressed per 100,000 person years or population respectively. The overall data estimates of age- and sex-specific prevalence and incidence from all studies were used in our modelling (see **Table S3** in **File S1**). A meta-regression-like epidemiological model in the form of a bubble graph was applied (done separately for males and females), adjusted for mean ages and the crude prevalence and incidence rates of stroke from all studies, with the size of the bubble corresponding to the given sample size. The fitted curve explaining the largest proportion of variance (best fit) was applied. From all data points, the median year of study was estimated, and the equations generated from the modelled curves were then separately used to estimate the new cases of stroke and number of stroke survivors at midpoints of the United Nation (UN) population 5-year age groups for the estimated median year. Africa populations were determined from the 2012 United Nations population demographics [22]. All statistical analyses were conducted on Stata 13.1 (Copyright 1985-2013 StataCorp LP).

## Results

### Systematic review

**Figure 1:**
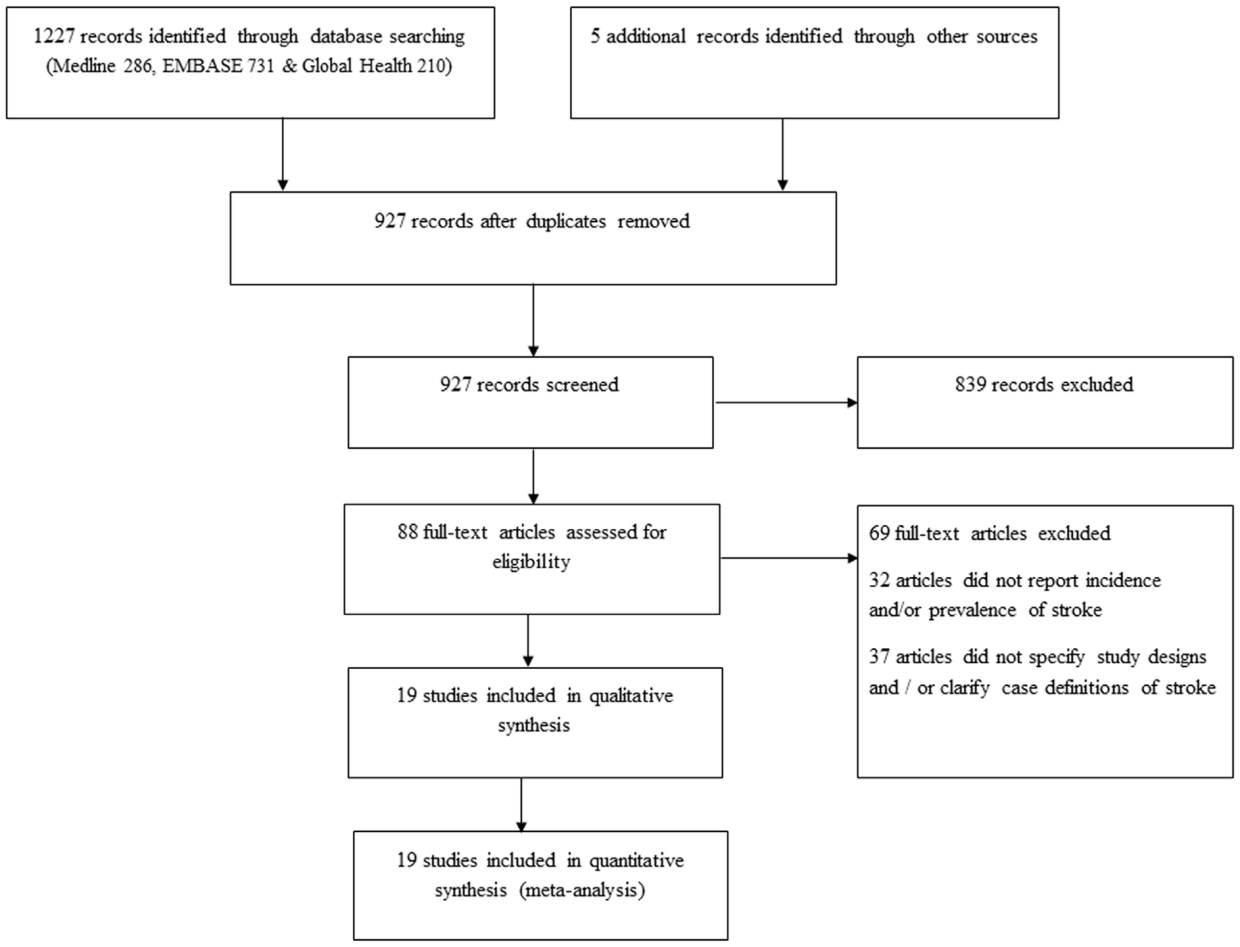
Flow diagram of search strategy.

The literature search returned 1227 publications from Medline (286), EMBASE (731) and Global Health (210). A further 5 studies were included from other sources (Google Scholar and reference lists of relevant publications). 927 studies remained after removing duplicates. On screening titles for relevance (stroke studies conducted primarily on African populations), 839 studies were excluded, giving a total of 88 full texts that were assessed. After applying the quality criteria, 69 studies were further excluded (32 articles did not provide numerical estimates on incidence and/or prevalence of stroke, and 37 articles did not clarify study designs and survey methodologies). A total of 19 studies were finally retained for the review (**Figure 1**).

### Study characteristics

The retained 19 studies [23]–[41] were conducted across the main regions of Africa (east, north, west and south), but with Northern Africa having the highest output (7 studies). 10 African countries were represented; Egypt and Nigeria ranked highest with 4 studies each, Libya, South Africa and Tanzania had two studies each, while Benin, Ethiopia, Mozambique, Tunisia and Zimbabwe had one study each. Most studies (84.2%) were completed within one year period and the median year of study from all data points was 2009. About 57.9% of studies were conducted in predominantly urban settings. The total sample size from all retained studies was over 6.3 million, with a mean and median sample size of 332,276.5 and 60,820 respectively. 14 studies were population-based, of which 8 were community-based door-to-door surveys and 2 studies each were based on demographic health surveys, population/community-based stroke registries and cross-sectional population-based surveys. There were 5 hospital-based studies with only one from a hospital-based stroke registry. Studies comply with the WHO case ascertainment or a modified definition, while some studies employed cranial computed tomography (CT) or magnetic resonance imaging (MRI) to confirm diagnosis (**Table 2**). Most studies were conducted on the entire study population with an overall mean age of 55.9 years. Across retained studies, age determination of subjects were determined from documented age-verification records, and in the absence of such, historical landmarks were employed.

**Table 2:** Overall characteristics of retained studies.

Country, Location, Setting	Author, Year	Study period	Survey method	Case definition
**EASTERN AFRICA**				
Ethiopia, Rural communities [23]	Tekle-Haimanot et al. 1990	1986–88	A door-to-door survey	WHO definition
Tanzania, Hai District, Rural [24]	Dewhurst et al. 2013	1 November 2009 and 31 July 2010	Point prevalence of stroke estimated from a cross-sectional two-phased community epidemiological survey.	WHO International Statistical Classification of Diseases and Related Health Problems 10th Revision (ICD-10)
Tanzania, Hai district & Dares Salaam, Mixed Rural and Urban [25]	Walker et al. 2010	2003–06	Stroke Incidence measured in two well defined demographic surveillance sites (DSS) over a 3-year period. Patients who had first-ever or recurrent strokes were included. Patients were excluded in suspected cases of infection or a space-occupying lesion	WHO definition
**NORTHERN AFRICA**				
Egypt, Al Kharga district, Mixed [26]	Farghaly et al. 2013	June 1, 2005 to May 31, 2009	A door-to-door screening including every door was carried out using a standardized questionnaire	WHO definition
Egypt, Al Quseir. Urban [27]	El Tallawy et al. 2013	July 1, 2009 to January 31, 2012	A door-to-door survey of every household in the district	WHO definition
Egypt, Assuit, Urban [28]	Khedr et al. 2013	January 1 2010 - December 31 2010	Community-based study using a three phase door-to-door survey	WHO definition
Egypt, Sohag, Mixed Urban and Rural [29]	Kandil et al. 2006	January 1st 1992 to April 30, 1993	Multistage, systematic random sampling using a door-to-door survey	WHO definition. Diagnosis confirmed by CT scan and other laboratory investigations creatinine
Libya, Benghazi, Urban [30]	El Zunni et al. 1995	January 1991 to December 1993	Survey conducted on patients referred from the walk-in polyclinics to the four university hospitals and to a rehabilitation center for the handicapped.	Cranial CT scan was performed on all cases within the first week of onset of stroke
Libya, Benghazi, Urban [31]	Ashok et al. 1986	November 1, 1983 and October 31, 1984	Hospital-based survey conducted on referred patients with neurological problems	Cranial CT was performed on cases within the first week of onset of stroke. Survey based on the US National Survey of Stroke guidelines
Tunisia, Kelibia, Mixed Urban and Rural [32]	Attia Romdhane et al. 1993	1985	Population-based survey	WHO definition and neurologic tool
**SOUTHERN AFRICA**				
Mozambique, Maputo, Urban [33]	Damasceno et al. 2010	August 1, 2005, to July 31, 2006	Hospital-based survey using the STEPS Stroke questionnaire. Both first-ever and recurrent stroke events were registered	WHO definition: “a focal (or at times global) neurological impairment of sudden onset, and lasting more than 24 hours (or leading to death), and of presumed vascular origin.”
South Africa, Agincourt Health and Population Unit, Limpopo province, Rural [34]	Connor et al. 2004	August 2001- October 2002	Point prevalence of stroke survivors measured through door-to-door demographic health survey. Person's first-ever-in-a-lifetime event was recorded	WHO definition: “rapidly developing signs of focal (or global) disturbance of cerebral function, leading to death or lasting longer than 24 hours, with no apparent cause other than vascular”. Person's first-ever-in-a-lifetime event was recorded
South Africa, Atteridgeville and Mamelodi suburban areas of Pretoria, Urban [35]	Rosman 1986	May 1 1984-April 30 1985	Prospective hospital-based survey. Included all strokes (first-ever and recurrent)	Diagnosis confirmed by cranial CT
Zimbabwe, Harare, Urban [36]	Matenga 1997	Jan- Dec 1991	A hospital-based stroke registry survey. Only first-ever strokes were included	Stroke was defined according to the WHO definition. None had CT
**WESTERN AFRICA**				
Benin, Cotonou, Urban [37]	Cossi et al. 2012	September 15, 2008- May 15, 2009	A three-phase door-to-door study was performed	Diagnosis of stroke was confirmed by CT scan evaluation
Nigeria, Ibadan, Urban [38]	Osuntokun et al. 1979	1973–75	Population-based stroke registry survey	WHO definition
Nigeria, Igbo-Ora, Rural [39]	Osuntokun et al. 1987	1982	Community-based door-to door survey	WHO definition
Nigeria, Lagos, Urban [40]	Danesi et al. 2013	January 1st and December 31^st^ 2007	Prospective community-based stroke registry enrolling hospitalized and non-hospitalized first-ever in a lifetime stroke cases presenting at all health facilities	Stroke was defined using the WHO clinical criteria ‘sudden onset of focal neurological deficit lasting longer than 24 h or leading to death with no other cause other than a vascular event'
Nigeria, Lagos, Urban [41]	Danesi et al. 2007	June 1, 2005, and May 30, 2006	Population-based, door-to-door survey using modified WHO questionnaire	Stroke defined as “a focal (or at times global) neurological impairment of sudden onset, and lasting more than 24 hours (or leading to death), and of presumed vascular origin.”

CT: computed tomography, ICD: International Classification of Disease, WHO: World Health Organization

### Pooled estimates of reported crude stroke incidence and prevalence rates

**Figure 2:**
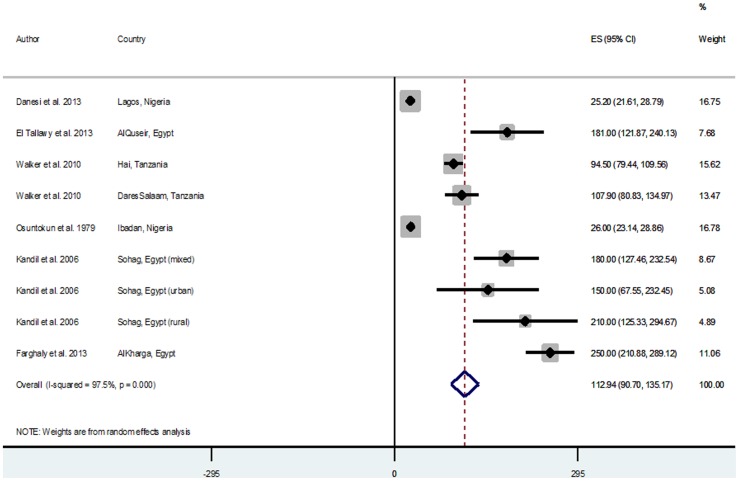
Pooled crude incidence rates of stroke from population-based studies.

**Figure 3:**
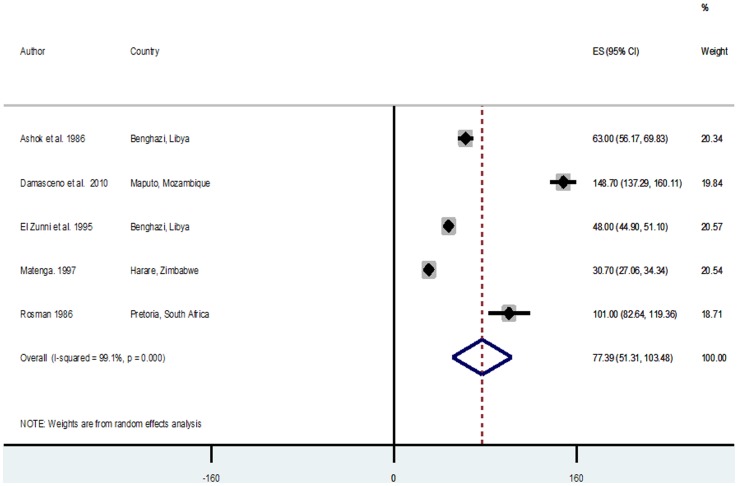
Pooled crude incidence rates of stroke from hospital-based studies.

**Figure 4:**
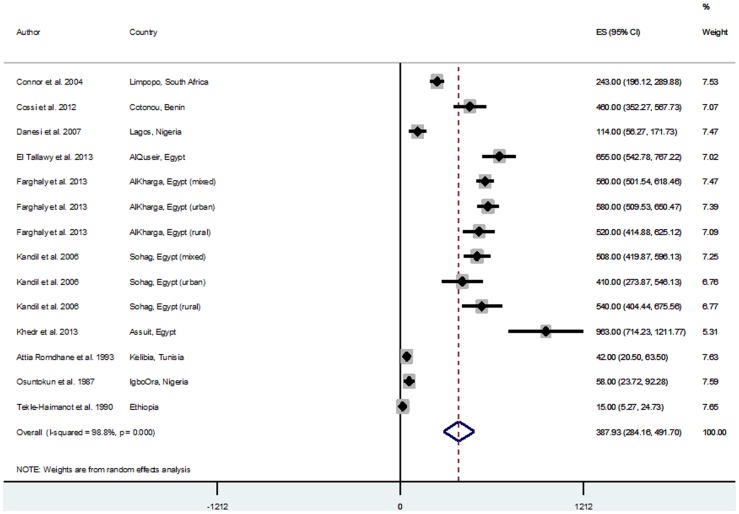
Pooled crude prevalence rates of stroke survivors from population-based studies.

Across studies reporting crude incidences of stroke, there were 6 population/community-based and 5 hospital-based studies. Population-based incidence rates were generally higher ranging from 25.2/100,000 person years (py) and 26.0/100,000 py in Lagos and Ibadan Nigeria in 2007 and 1979 respectively [38], [40], to 250/100,000 py in Al-Kharga Egypt in 2007 [26]. The hospital-based studies reported lower incidence rates ranging from 30/100,000 py in Harare Zimbabwe in 1991 [36], to 148.7/100,000 py in Maputo Mozambique in 2006 [33] (**Table 3**). The random effect meta-analysis of population-based incidence rates was 112.94/100,000 py (95% CI = 90.7–135.17, I^2^ = 97.5%, p = 0.000) (**Figure 2**). The hospital-based meta-analysis was lower with a pooled estimate of 77.39/100,000 py (95% CI = 51.31–103.48, I^2^ = 99.1%, p = 0.000) (**Figure 3**).There were 11studies (all population/community-based) reporting crude prevalences of stroke survivors with prevalence rates ranging from 15/100,000 population in Ethiopia in 1988 [23], to 963/100,000 population in 2010 [28] (**Table 4**). Random effect meta-analysis yielded a pooled prevalence rate of 387.93/100,000 population (95% CI = 284.16–491.70, I^2^ = 98.8%, p = 0.000) (**Figure 4**). A Tanzanian study reported a prevalence of 2300/100,000 among people aged 70 years above in Hai district in 2010 [24], this was not included in the meta-analysis as other studies were mostly based on the general population with mean age ranging between 50 and 60 years (**Table 4**).

**Table 3:** Summary of data from studies reporting crude incidence of stroke.

Author	Year	Age (years)	Cases (All)	Sample size (All)	Incidence/100000 py (All)	Cases (Male)	Sample size (Male)	Incidence/100000 py (Men)	Cases (Female)	Sample size (Female)	Incidence/100000 py (Women)
**POPULATION/COMMUNITY-BASED**											
**Danesi et al. 2013**	2007	All	189	750000	25.2	118	417000	28.3	71	333000	21.3
**El Tallawy et al. 2013**	2012	20+	36	19848	181	21	9916	212	15	9932	150
**Walker et al. 2010a**	2006	All	453	159814	94.5	532	71916	106.7	10	87898	76.7
**Walker et al. 2010b**	2006	All	183	56517	107.9	266	25433	115.2	122	31084	99.7
**Osuntokun et al. 1979**	1975	All	318	1223077	26	229	538462	25	89	684615	13
**Kandil et al. 2006a**	1993	All	39	25000	180	21	21000	100	18	21176	85
**Kandil et al. 2006b**	1993	All	11	8464	150	7	7778	90	4	7547	53
**Kandil et al. 2006c**	1993	All	20	11228	210	9	9278	97	11	9244	119
**Farghaly et al. 2013**	2007	All	156	62583	250	86	32165	270	70	30418	230
**HOSPITAL-BASED**											
**Ashok et al. 1986**	1984	15+	329	518745	63	184	267590	69	145	251155	58
**Damasceno et al. 2010**	2006	15+	651	437794	148.7	342	197007	173.6	309	240787	128.3
**El Zunni et al. 1995**	1993	15+	921	1918750	48	379	1196154	52	322	722596	42
**Matenga. 1997**	1991	All	273	889250	30.7	142	478114	29.7	131	411136	32
**Rosman 1986**	1985	20+	116	114931	101	65	60343	108	51	54588	93

Walker et al. 2010a: Hai district (rural setting), Walker et al. 2010b: Dares Salaam district (urban setting)

**Table 4:** Summary of data from studies reporting crude prevalence of stroke survivors (all population/community-based).

Author, year	Year	Age (years)	Cases (All)	Sample size (All)	Prevalence/100000 (All)	Cases (Men)	Sample size (Men)	Prevalence/100000 (Men)	Cases (Women)	Sample size (Female)	Prevalence/100000 (Men)
**Connor et al, 2004**	2002	15+	103	42378	243	37	20042	185	66	22336	296
**Cossi et al. 2012**	2009	15+	70	15155	460	38	6293	610	32	8862	360
**Danesi et al. 2007**	2006	All	15	13127	114	11	7295	151	4	5832	69
**Dewhurst et al. 2013** *	2010	70+	51	2232	2300	29	976	2971	22	1256	1752
**El Tallawy et al. 2013**	2012	All	130	19848	655	85	9916	860	48	9932	480
**Farghaly et al. 2013a**	2009	All	351	62583	560	196	32165	610	155	30418	510
**Farghaly et al. 2013b**	2009	All	257	44600	580	142	22908	620	115	21692	530
**Farghaly et al. 2013c**	2009	All	94	17983	520	54	9257	580	40	8726	458
**Kandil et al. 2006a**	1993	All	127	25000	508	65	12500	520	62	12500	490
**Kandil et al. 2006b**	1993	All	35	8464	410	20	4348	460	15	4116	470
**Kandil et al. 2006c**	1993	All	61	11228	540	29	5686	510	32	5542	570
**Khedr et al. 2013**	2010	All	57	5920	963	36	3066	1174	21	2854	736
**Attia Romdhane et al. 1993**	1985	All	15	34874	42	-	-	-	-	-	-
**Osuntokun et al. 1987**	1982	All	11	18954	58	-	-	-	-	-	-
**Tekle-Haimanot et al. 1990**	1988	20–85	9	60820	15	-	-	-	-	-	-

*not included in meta-analysis, a: mixed setting, b: urban setting, c: rural setting.

### Modelled estimates of stroke incidence and prevalence rates in Africa

Based on the UN population demographics and bubble graphs derived from all data points, incident cases and number of stroke survivors were estimated for the year 2009, which was our estimated median year of study.

**Figure 5:**
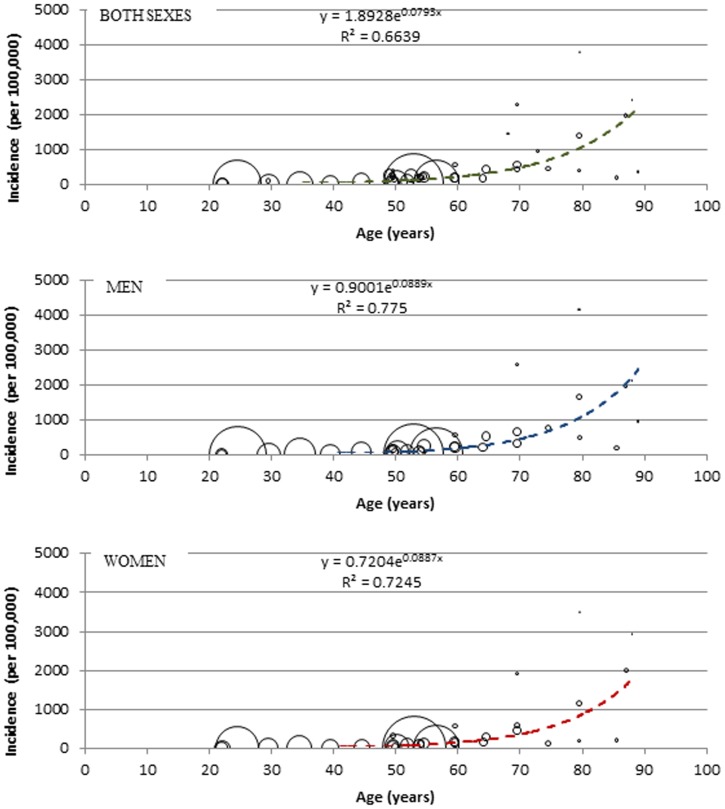
Bubble graph showing relationship between age and crude incidence of stroke, with size of bubble corresponding to sample size.

There were over 483 thousand new cases of stroke in Africa in 2009 among people aged 15 years or more equivalent to 81.2 (13.2–94.9)/100,000 py, with about 305 thousand and over 178 thousand new cases of stroke equivalent to 103.3 (20.7–109.2)/100,000 py and 59.5 (6.9–84.3)/100,000 py among men and women, respectively (**Table 5** and **Figure 5**). Comparable figures for the year 2010 and 2013 based on the same incidence rates would amount to 496 (80.6–579.7) and 535 (87.0–625.3) thousand new stroke cases respectively, suggesting an increase of 10.8% between 2009 and 2013 that is attributable to growth and ageing of the African population alone.

**Table 5:** Estimated new stroke cases and incidence rates in Africa in 2009 (estimates derived from bubble graph model and United Nations population figures).

Age (years)	Both sexes		Men		Women	
	Incidence (per 100,000 py) = 1.8928e^0.0793x^	Stroke cases (000)	Incidence (per 100,000 py) = 0.9001e^0.0889x^	Stroke cases (000)	Incidence (per 100,000 py) = 0.7204e^0.0887x^	Stroke cases (000)
**15–19**	7.3	7.744	4.1	6.028	3.3	1.715
**20–24**	10.8	10.312	6.4	7.910	5.1	2.402
**25–29**	16.1	13.131	9.9	9.919	7.9	3.212
**30–34**	23.9	16.034	15.5	11.933	12.3	4.101
**35–39**	35.6	19.141	24.1	14.009	19.2	5.132
**40–44**	52.9	23.261	37.7	16.668	29.9	6.593
**45–49**	78.7	29.061	58.7	20.316	46.6	8.744
**50–54**	116.9	36.059	91.6	24.532	72.6	11.527
**55–69**	173.8	43.290	142.9	28.622	113.1	14.668
**60–64**	258.4	50.181	222.9	32.166	176.2	18.016
**65–69**	384.2	54.939	347.6	33.881	274.5	21.058
**70–74**	571.2	57.775	542.2	34.087	427.7	23.688
**75–79**	849.1	52.338	845.6	29.437	666.4	22.901
**80+**	1601.3	69.783	1722.0	35.059	1355.0	34.724
**Total 15+**	81.2 (13.2–94.9)	483.050	103.3 (20.7–109.2)	304.567	59.5 (6.9–84.3)	178.482

py: person years, x = midpoint of age group.

**Figure 6:**
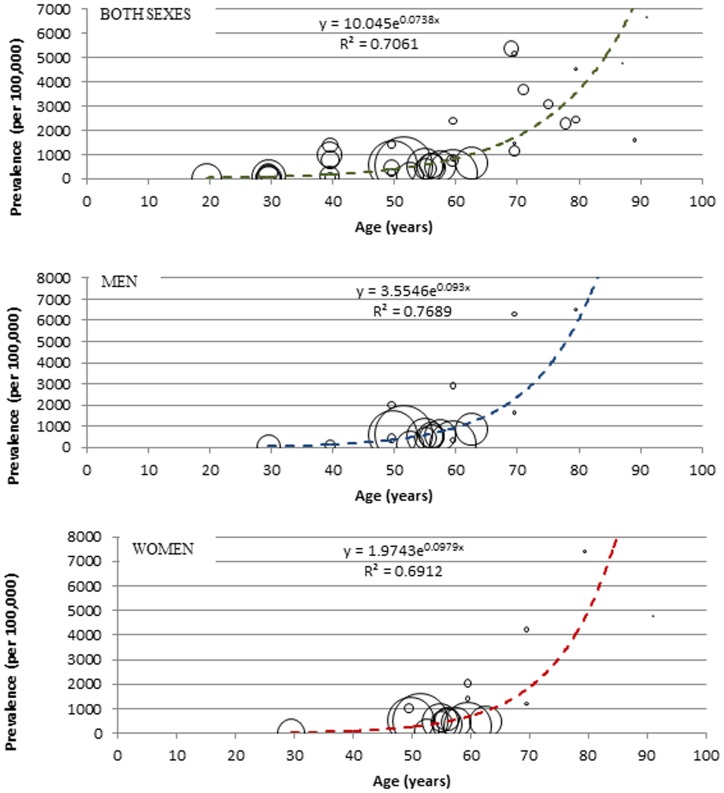
Bubble graph showing relationship between age and crude prevalence of stroke survivors, with size of bubble corresponding to sample size.

The estimated number of stroke survivors in Africa in 2009 was 1.89 million among people aged 15 years or more with a prevalence of 317.3 (314.0–748.2)/100000 population. There were about 990 thousand and 898 thousand stroke survivors equivalent to 335.5 (302.3–702.7)/100,000 and 299.3 (268.4–579.0)/100,000 among men and women, respectively (**Table 6** and **Figure 6**). Based on the same prevalence rates, comparable figures for the year 2010 and 2013 would amount to 1.94 (1.90–4.57) and 2.09 (2.06–4.93) million stroke survivors respectively, also suggesting an increase of 9.6% between 2009 and 2013 that is attributable to growth and ageing of the African population alone.

**Table 6:** Estimated number of stroke survivors and prevalence in Africa in 2009 (estimates derived from bubble graph model and United Nations population figures).

Age (years)	Both sexes		Men		Women	
	Prevalence (per 100,000) = 10.045e^0.0738x^	Stroke cases (000)	Prevalence (per 100,000) = 3.5546e^0.093x^	Stroke cases (000)	Prevalence (per 100,000) = 1.9743e^0.0979x^	Stroke cases (000)
**15–19**	35.2	14.747	17.3	9.250	10.4	5.497
**20–24**	50.9	21.209	27.5	13.149	17.0	8.060
**25–29**	73.8	29.183	43.9	17.899	27.8	11.284
**30–34**	106.6	38.546	69.7	23.461	45.3	15.085
**35–39**	154.1	49.752	110.9	29.985	73.9	19.768
**40–44**	222.9	65.282	176.7	38.691	120.5	26.591
**45–49**	322.4	88.022	281.3	51.095	196.7	36.927
**50–54**	466.2	117.911	447.8	66.938	320.9	50.973
**55–69**	674.3	152.945	712.8	85.030	523.5	67.914
**60–64**	975.2	191.641	1134.8	104.301	854.1	87.341
**65–69**	1410.5	226.648	1806.7	119.754	1393.4	106.894
**70–74**	2039.9	257.559	2876.2	131.654	2273.3	125.905
**75–79**	2950.3	252.359	4579.0	124.904	3708.9	127.455
**80+**	5324.5	381.003	9635.8	172.991	8116.9	208.012
**Total 15+**	317.3 (314.0–748.2)	1886.806	335.5 (302.3–704.7)	989.099	299.3 (268.4–579.0)	897.708

x = midpoint of age group.

## Discussion

Some systematic reviews have been published on the burden of stroke in Africa but without a continent-wide estimate of stroke incidence and prevalence rates [7], [42]. There are also global reviews of stroke with few studies on Africa population included [13], [43], [44]. For example, in a systematic review of 56 population-based studies globally, only one African site (Ibadan, Nigeria) was considered. The result from this survey may not necessarily reflect the overall burden of stroke in Africa [13]. However, to the best knowledge, this study provides the first continent-wide estimate of the incidence and prevalence rates of stroke in Africa. The estimates were strictly based on a simple statistical analysis with appropriate consideration of reported mean ages and sample sizes from individual studies. Moreover, having applied the UN population demographics in our final model, the current estimates fairly reflect the, albeit very limited, available published data on incidence and prevalence rates of stroke in Africa, and may help policymakers across several African countries institute effective public health response to the growing burden.

Generally, crude incidences of stroke from population/community based-studies were higher with the two low incidence rates recorded obtained from population-based stroke registries in Nigeria [38], [40] (**Table 3**). Despite over 3 decades of potential improvement in stroke registration between the two studies (1975–2007), the low incidence rates may still be indicative of incompleteness of stroke registries in many Africa settings, and that data obtained from these registries may be unreliable and inappropriate for estimation of stroke burden. In contrast, in many high income countries where there is active registration of stroke cases, population-based stroke registries have been reliable sources of data for estimation of stroke incidences [7], [45]. In this study, the pooled crude incidence rate from community-based studies was higher at 112.9 per 100,000 person years compared to 77.4 from hospital-based studies. The difference suggests a likely under-estimation of stroke incidences from hospital-based studies, which has also been observed by some studies, particularly due to the very few stroke cases presenting to standard health facilities [2]. The observed prevalence rates of stroke survivors were generally high (all prevalence studies were population-based) with a pooled crude prevalence rate of 387.9/100,000 population. The low prevalence rate recorded in Ethiopia in 1988 may not be unconnected with the high mortality rates from stroke, which has generally been reported in many parts of Africa [23], [46]. Moreover, the Ethiopian study was broadly a survey of neurological disorders in the community, which could possibly imply that active case recognition of specific stroke cases may be less rigorous.

The modelling showed a rising incidence and prevalence rates of stroke with increasing age and higher figures recorded among men, which is in keeping with several research findings on stroke burden [24]. Over 483 thousand new cases of stroke with an incidence rate of 81.2/100,000 py (men 103.3, women 59.5), and about 1.89 million stroke survivors with a prevalence rate of 317.3/100000 population (men 335.5, women 299.3), both among people aged 15 years or more were estimated in 2009. A report in 2004 suggested that that about 8% of all first-ever strokes (about 5 million) occurred in Africa and 5% of over 30 million stroke survivors worldwide were in Africa [5], [11], and this amounts to about 400 thousand new stroke cases and 1.5 million stroke survivors. A systematic review in sub-Saharan Africa on studies published between 1966–2006 showed age-standardized prevalence rates of 114–315/100,000 populations and 154–281/100,000 population among men and women, respectively [7]. Another review in 2006 showed that the prevalence of stroke survivors ranges from 200–300/100,000 population in sub-Saharan Africa, and incidence rates ranges from 15–68/100,000 py [42]. These figures are comparable with the current estimates, which further underpins a near representation of the burden of stroke in Africa. The minor differences may probably be due to the study periods, age groups, fewer data-points, focus on sub-Saharan Africa, and the fact that these were largely qualitative reviews and not based on a detailed statistical synthesis. However, according to the 2014 GBD estimates by Feigin and colleagues, over a 100% increase in the total number of new stroke cases and stroke survivors was recorded between 1990 and 2010 in LMIC, with an estimated incidence rate of 281.1/100,000 py and prevalence rate of 393.4/100,000 population in 2010 [1]. While it is understandable that not all LMIC have contextual similarities with African countries and direct comparisons may be inappropriate, it may however still be logical to conclude that the estimates from this study reflect the current stroke burden in many LMIC.

While this study aimed to provide an evidence-based continent-wide estimation of stroke incidence and prevalence rates in Africa through simple statistical analysis, the study has some important limitations. In particular the low research output and quality of selected studies from Africa constrained the overall analysis. There were 19 studies covering only 10 countries in Africa with an overall sample size of over 6.3 million. Moreover, stroke case ascertainment were not well defined across some studies, and this has been documented in some reviews [45]. Due to these limited data and with some full texts assessed showing evidence of a detailed epidemiological exercise, an inclusion of some of these studies in our final analysis was allowed. In addition, some reported stroke incidence data were based on both first stroke and recurrent stroke events, and not all reported prevalence rates were strictly based on stroke survivors. However, having ensured all studies showed evidence of a degree of epidemiological survey rigour, and all extracted data points included in our modelling (refer to **Table 2**, and **Table S3** in **File S1**), the current estimates could still give a near representation of the burden of stroke in Africa.

### Public health response to stroke in Africa

The prevention of stroke and many non-communicable diseases in Africa has been affected mainly by weak health systems and poor government response [46]. To date, the priorities of many African countries remain infectious diseases: mainly HIV/AIDS, malaria and tuberculosis [3], [47], despite the availability of affordable and cost effective stroke prevention initiatives [48], [49]. For example, Walker and colleagues reported that African countries do not have national strategies to address smoking, alcohol, physical inactivity and unhealthy diets including reducing salt and fat contents of processed foods [48]; and stroke units, where the awareness on these risk factors could have been raised, are rarely available [12], [50]. The INTERSTROKE study findings show that hypertension is the main risk factor of all stroke subtypes with odds of about 2.64 [8], this is more prominent among young Africans who present with stroke unaware of their high blood pressure status [15]. Truelsen argues that the prevalence of stroke in Africa might increase due to substantial changes in major stroke risk factors in the presence of a biased focus on the prevention and control of infectious diseases, at the expense of many NCDs [51].

The diagnosis of stroke in many African settings remain a huge challenge [42]. Some hospital surveys in sub-Saharan Africa have shown that CT scans are only conducted on less than half of patients presenting with stroke, and this is mainly among those that can afford it [42], [52]. In fact, experts have reported that the unavailability and/or high costs of cranial CT imaging in many parts of Africa have limited information on the pathologic profiles of different stroke types in the continent, with this often affecting the diagnosis, treatment and the overall management of the disease [52]. Reports further show that in some areas with better population-wide access to CT scans, as the case in Tanzania, Ghana, and the Medical University of Southern African (MEDUNSA) Stroke Data Bank (MSDB), there have been improvements in diagnosis of stroke, with varying cases of ischaemic and haemorrhagic strokes reported [53]–[55]. Moreover, the challenges of appropriately distinguishing first from recurrent stroke episodes have also affected stroke case ascertainment, especially during epidemiological surveys [7] Neurologists have noted the importance of proper planning during community surveys, active registration and follow-up of new stroke cases identified, and training and re-training of health workers on stroke diagnosis [17]; arguing that the absence of these in many African settings have resulted in increased number of stroke cases in the community who have never had contact with standard health facilities, and inability to categorize these as first or recurrent strokes, including relating such existing stroke cases to a particular period during surveys [7], [17].

As noted earlier, a systematic review has suggested that income is a strong predictor of stroke risk and fatalities [6]. For example, the average cost of a cranial CT in Ugandan was approximately $60 USD between 2000 and 2010 [2]. This is expensive in most African population groups where many still live below the poverty index of less than $1.25/day [56]. The high cost of health services in the absence of an effective health insurance schemes and adequate resources allocated for stroke prevention and management has affected healthcare seeking behaviour in some African settings [7], [57]. Many stroke patients have been managed at home due to lack of hospital funds, with only few presenting to standard health facilities several days after the onset of symptoms having tried low-priced under-resourced clinics [2]. For those who manage to get to standard health facilities, there are also challenges arising from poor quality of care, as several studies have reported massive gaps exist in the management of acute stroke in Africa compared to many high income countries [58], [59].

The unavailability of data with low research output has been a major setback in the management of stroke in Africa [51]. Experts have reported that reliable data from which evidence-based policy decisions can be made are sparse in Africa [60]. Many have argued that no study in Africa can be regarded as an ideal stroke study [17], adding that there were no proper stroke registries and demographic health surveys which invariably limit active registration, follow-up of cases and conduct of community-based studies [45]. Based on the current findings, hospital-based studies and door-to-door surveys were mainly conducted in Africa and despite the rigour of these few epidemiological surveys, gaps have been identified with regards to case ascertainment and study protocols [46].

Our findings suggest an increasing burden of stroke in Africa. However, with the current low availability of data, there is still need for more research on stroke, and related vascular disease risk factors to appropriately quantify this burden. An investment in research capacity, basically to conduct and fund higher quality research may help raise awareness on stroke burden in Africa. An awareness and fair understanding of stroke burden and disease pattern in Africa may further prompt appropriate policy response and scale up current intervention programmes.

## Supporting Information

File S1 **Table S1.** Search terms (EMBASE). **Table S2.** Search terms (Global Health). **Table S3.** All data points employed in modelling.
(DOC)

Checklist S1 PRISMA Checklist.
(DOC)
