# Supplementary material for: An Estimate of the Incidence and Prevalence of Stroke in Africa: A Systematic Review and Meta-Analysis
Source: PLoS One. 2014 Jun 26;9(6):e100724. doi: 10.1371/journal.pone.0100724 (PMC4072632; doi:10.1371/journal.pone.0100724)
Supplement: File S1 — Table S1. Search terms (EMBASE). Table S2. Search terms (Global Health). Table S3. All data points employed in modelling. (DOC) [file pone.0100724.s001.doc]

**SUPPORTING INFORMATION**

**Table S1. Search terms (EMBASE)**

| **#** | | **Searches** |
| --- | --- | --- |
|  | | africa/ or africa, northern/ or algeria/ or egypt/ or libya/ or morocco/ or africa, central/ or cameroon/ or central african republic/ or chad/ or congo/ or "democratic republic of the congo"/ or equatorial guinea/ or gabon/ or africa, eastern/ or burundi/ or djibouti/ or eritrea/ or ethiopia/ or kenya/ or rwanda/ or somalia/ or sudan/ or tanzania/ or uganda/ or africa, southern/ or angola/ or botswana/ or lesotho/ or malawi/ or mozambique/ or namibia/ or south africa/ or swaziland/ or zambia/ or zimbabwe/ or africa, western/ or benin/ or burkina faso/ or cape verde/ or cote d'ivoire/ or gambia/ or ghana/ or guinea/ or guinea-bissau/ or liberia/ or mali/ or mauritania/ or niger/ or nigeria/ or senegal/ or sierra leone/ or togo/ |
|  | | exp vital statistics/ or exp incidence/ |
|  | | (incidence* or prevalence* or morbidity or mortality).tw. |
|  | | (disease adj3 burden).tw. |
|  | | exp "cost of illness"/ |
|  | | exp quality-adjusted life years/ |
|  | | QALY.tw. |
|  | | Disability adjusted life years.mp. |
|  | | (initial adj2 burden).tw. |
|  | | exp risk factors/ |
|  | | 2 or 3 or 4 or 5 or 6 or 7 or 8 or 9 or 10 |
|  | | stroke/ or brain infarction/ or brain stem infarctions/ or cerebral infarction/ or stroke, lacunar/ |
|  | | cerebrovascular accident [Used For] accident, cerebrovascular, acute focal cerebral, vasculopathy, apoplectic stroke, apoplexia, apoplexy, blood flow disturbance, brain, brain accident, brain attack, brain blood flow disturbance, brain insult, brain insultus, brain ischemic attack, brain vascular accident, cerebral apoplexia, cerebral insult, cerebral stroke, cerebral vascular accident, cerebral vascular insufficiency, cerebro vascular accident, cerebrovascular arrest or failure or insufficiency or insult or trauma, cerebrum vascular accident, cryptogenic stroke, CVA, ischaemic seizure, ischemic cerebral attack, ischemic seizure, stroke |
|  | | cerebrovascular disease.mp. (broader terms) |
|  | | CVA.mp. |
|  | | 12 or 13 or 14 or 15 |
|  | | 1 and 11 and 16 |
|  |  |  |

**Table S2. Search terms (Global Health)**

| **#** | **Searches** |
| --- | --- |
|  | africa/ or africa, northern/ or algeria/ or egypt/ or libya/ or morocco/ or africa, central/ or cameroon/ or central african republic/ or chad/ or congo/ or "democratic republic of the congo"/ or equatorial guinea/ or gabon/ or africa, eastern/ or burundi/ or djibouti/ or eritrea/ or ethiopia/ or kenya/ or rwanda/ or somalia/ or sudan/ or tanzania/ or uganda/ or africa, southern/ or angola/ or botswana/ or lesotho/ or malawi/ or mozambique/ or namibia/ or south africa/ or swaziland/ or zambia/ or zimbabwe/ or africa, western/ or benin/ or burkina faso/ or cape verde/ or cote d'ivoire/ or gambia/ or ghana/ or guinea/ or guinea-bissau/ or liberia/ or mali/ or mauritania/ or niger/ or nigeria/ or senegal/ or sierra leone/ or togo/ |
|  | exp vital statistics/ or exp incidence/ |
|  | (incidence* or prevalence* or morbidity or mortality).tw. |
|  | (disease adj3 burden).tw. |
|  | exp "cost of illness"/ |
|  | exp quality-adjusted life years/ |
|  | QALY.tw. |
|  | Disability adjusted life years.mp. |
|  | (initial adj2 burden).tw. |
|  | exp risk factors/ |
|  | 2 or 3 or 4 or 5 or 6 or 7 or 8 or 9 or 10 |
|  | stroke/ or brain infarction/ or brain stem infarctions/ or cerebral infarction/ or stroke, lacunar/ broader terms [derrame cerebral](http://ovidsp.tx.ovid.com.ezproxy.is.ed.ac.uk/sp-3.12.0b/ovidweb.cgi?S=IJMKFPCIJLDDEJPLNCMKOEGCAAEGAA00&Controlled+Vocabulary=thes+derrame+cerebral&) |
|  | cerebrovascular accident.mp. |
|  | cerebrovascular disorders [used for] [disturbios cerebrovasculares](http://ovidsp.tx.ovid.com.ezproxy.is.ed.ac.uk/sp-3.12.0b/ovidweb.cgi?S=IJMKFPCIJLDDEJPLNCMKOEGCAAEGAA00&Controlled+Vocabulary=thes+disturbios+cerebrovasculares&), [trastornos cerebrovasculares](http://ovidsp.tx.ovid.com.ezproxy.is.ed.ac.uk/sp-3.12.0b/ovidweb.cgi?S=IJMKFPCIJLDDEJPLNCMKOEGCAAEGAA00&Controlled+Vocabulary=thes+trastornos+cerebrovasculares&), broader terms [brain diseases](http://ovidsp.tx.ovid.com.ezproxy.is.ed.ac.uk/sp-3.12.0b/ovidweb.cgi?S=IJMKFPCIJLDDEJPLNCMKOEGCAAEGAA00&Controlled+Vocabulary=thes+brain+diseases&) |
|  | CVA.mp. |
|  | 12 or 13 or 14 or 15 |
|  | 1 and 11 and 16 |

**Table S3. All data points employed in modelling**

| Study Id | Author, year | Year | Age | Cases (All) | Denominator (All) | Prevalence(Incidence) /100000 (All) | Cases (Men) | Denominator (Men) | Prevalence(Incidence) /100000 (Men) | Cases (Women) | Denominator (Women) | Prevalence(Incidence) /100000 (Women) |
| --- | --- | --- | --- | --- | --- | --- | --- | --- | --- | --- | --- | --- |
| PREVALENCE DATA | | | | | | | | | | | | |
| 1 | Connor et al. 2004 | 2001-02 | all 15+ | 103 | 42378 | 243 | 37 | 20042 | 185 | 66 | 22336 | 296 |
|  |  |  | 15-24 | 2 | 15358 | 13 |  |  |  |  |  |  |
|  |  |  | 25-34 | 6 | 10315 | 58 |  |  |  |  |  |  |
|  |  |  | 35-44 | 8 | 6855 | 117 |  |  |  |  |  |  |
|  |  |  | 45-54 | 21 | 4340 | 484 |  |  |  |  |  |  |
|  |  |  | 55-64 | 19 | 2497 | 761 |  |  |  |  |  |  |
|  |  |  | 65-74 | 22 | 1925 | 1143 |  |  |  |  |  |  |
|  |  |  | 75-84 | 22 | 901 | 2442 |  |  |  |  |  |  |
|  |  |  | 85+ | 3 | 187 | 1604 |  |  |  |  |  |  |
| 3 | Cossi et al. 2012 | 2008-09 | all 15+ | 70 | 15155 | 460 | 38 | 6293 | 610 | 32 | 8862 | 360 |
|  |  |  | 15-44 | 4 | 12580 | 30 | 3 | 5300 | 60 | 1 | 7279 | 10 |
|  |  |  | 45-54 | 17 | 1212 | 1400 | 9 | 443 | 2030 | 8 | 769 | 1040 |
|  |  |  | 55-64 | 19 | 799 | 2380 | 9 | 309 | 2910 | 10 | 490 | 2040 |
|  |  |  | 65-74 | 18 | 348 | 5170 | 10 | 159 | 6290 | 8 | 189 | 4230 |
|  |  |  | 75-84 | 10 | 134 | 7460 | 6 | 50 | 12000 | 4 | 84 | 7410 |
|  |  |  | 85+ | 2 | 30 | 6670 | 1 | 9 | 11100 | 1 | 21 | 4760 |
| 5 | Danesi et al. 2007 | 2005-06 | all 0+ | 15 | 13127 | 114 | 11 | 7295 | 151 | 4 | 5832 | 69 |
|  |  |  | 35-44 | 1 | 1802 | 56 | 1 | 999 | 100 | 0 | 803 | 0 |
|  |  |  | 45-54 | 3 | 1219 | 246 | 3 | 704 | 426 | 0 | 515 | 0 |
|  |  |  | 55-64 | 4 | 492 | 813 | 1 | 284 | 357 | 3 | 211 | 1421 |
|  |  |  | 65-74 | 3 | 203 | 1478 | 2 | 120 | 1667 | 1 | 83 | 1205 |
|  |  |  | 75-84 | 3 | 66 | 4545 | 3 | 46 | 6522 | 0 | 20 | 0 |
|  |  |  | 85+ | 1 | 21 | 4761 | 1 | 8 | 12500 | 0 | 13 | 0 |
| 7 | Dewhurst et al. 2013 | 2009-10 | 70+ | 51 | 2232 | 2300 |  | 976 |  |  | 1256 |  |
| 8 | El Tallawy et al. 2013 | 2009-12 | all 20+ | 130 | 19848 | 655 | 85 | 9916 | 860 | 48 | 9932 | 480 |
|  |  |  | 20-39 | 3 | 11664 | 26 |  |  |  |  |  |  |
|  |  |  | 40-59 | 49 | 6077 | 806 |  |  |  |  |  |  |
|  |  |  | 60+ | 78 | 2107 | 3702 |  |  |  |  |  |  |
| 12 | Farghaly et al. 2013a (El Tallawy 2010) | 2005-09 | all 0+ | 351 | 62583 | 560 | 196 | 32165 | 610 | 155 | 30418 | 510 |
|  | Farghaly et al. 2013b (El Tallawy 2010) | 2005-09 | all 0+ | 257 | 44600 | 580 | 142 | 22908 | 620 | 115 | 21692 | 530 |
|  | Farghaly et al. 2013c (El Tallawy 2010) | 2005-09 | all 0+ | 94 | 17983 | 520 | 54 | 9257 | 580 | 40 | 8726 | 460 |
|  |  |  | 20-39 | 18 | 20334 | 90 |  |  |  |  |  |  |
|  |  |  | 40-59 | 114 | 11545 | 990 |  |  |  |  |  |  |
|  |  |  | 60+ | 212 | 3937 | 5380 |  |  |  |  |  |  |
| 15  15  15 | Kandil et al. 2006a | 1992-93 | all 0+ | 127 | 25000 | 508 | 65 |  | 520 | 62 |  | 490 |
|  | Kandil et al. 2006b | 1992-93 | all 0+ | 35 | 8464 | 410 | 20 |  | 460 | 15 |  | 470 |
|  | Kandil et al. 2006c | 1992-93 | all 0+ | 61 | 11228 | 540 | 29 |  | 510 | 32 |  | 570 |
|  |  |  | 20-39 | 17 | 6629 | 260 |  |  |  |  |  |  |
|  |  |  | 40-59 | 56 | 4017 | 1390 |  |  |  |  |  |  |
|  |  |  | 60+ | 52 | 1688 | 3080 |  |  |  |  |  |  |
| 16 | Khedr et al. 2013 | 2010 | 0+ | 57 | 5920 | 963 | 36 | 3066 | 1174 | 21 | 2854 | 736 |
| 17 | Attia Romdhane et al. 1993 | 1985 | 0+ | 15 | 34874 | 42 |  |  |  |  |  |  |
| 18 | Osuntokun et al. 1987 | 1982 | 0+ | 11 | 18954 | 58 |  |  |  |  |  |  |
| 19 | Tekle-Haimanot et al. 1990 | 1988 | 20-85 | 9 | 60820 | 15 |  |  |  |  |  |  |
| INCIDENCE DATA | | | | | | | | | | | | |
| 6i | Danesi et al. 2013 | 2007 | all 0+ | 189 | 750000 | 25.2 | 118 | 417000 | 28.3 | 71 | 333000 | 21.3 |
|  |  |  | 25-34 | 9 | 148560 | 6.1 | 5 | 83400 | 6 | 4 | 65160 | 6.1 |
|  |  |  | 35-44 | 22 | 102860 | 21.4 | 15 | 57127 | 28.8 | 7 | 45733 | 15.3 |
|  |  |  | 45-54 | 36 | 69750 | 51.6 | 24 | 40446 | 59.4 | 12 | 29304 | 40.95 |
|  |  |  | 55-64 | 54 | 28610 | 188.7 | 36 | 16262 | 221.4 | 18 | 12348 | 145.8 |
|  |  |  | 65-74 | 50 | 11343 | 440.8 | 22 | 6680 | 329.3 | 28 | 4663 | 600.5 |
|  |  |  | 75-84 | 14 | 3501 | 399.9 | 12 | 2502 | 479.6 | 2 | 999 | 200.2 |
|  |  |  | 85+ | 4 | 1086 | 368.3 | 4 | 418 | 956.9 |  |  |  |
| 8i | El Tallawy et al. 2013 | 2012 | all 20+ | 36 | 19848 | 181 | 21 | 9916 | 212 | 15 | 9932 | 150 |
|  |  |  | 40-59 | 16 | 6077 | 263 |  |  |  |  |  |  |
|  |  |  | 60+ | 20 | 2107 | 950 |  |  |  |  |  |  |
| 11i | Walker et al. 2010a | 2006 | all 0+ | 453 | 159814 | 94.5 | 235 | 71916.3 | 106.7 | 10 | 87897.7 | 76.7 |
|  |  |  | 0-44 | 34 | 60216.7 | 9 | 17 | 21948.06 | 8 | 17 | 39132.21 | 9.9 |
|  |  |  | 45-54 | 32 | 38600.72 | 82.9 | 20 | 21905.81 | 91.3 | 12 | 15810.28 | 75.9 |
|  |  |  | 55-64 | 55 | 26712 | 205.9 | 30 | 12484.39 | 240.3 | 25 | 14068.66 | 177.7 |
|  |  |  | 65-74 | 116 | 20415.35 | 568.2 | 66 | 9776.329 | 675.1 | 50 | 10636.03 | 470.1 |
|  |  |  | 75-84 | 139 | 9981.33 | 1392.6 | 70 | 4175.365 | 1676.5 | 69 | 5996.35 | 1150.7 |
|  |  |  | 85+ | 77 | 3887.907 | 1980.5 | 32 | 1626.347 | 1967.6 | 45 | 2254.17 | 1996.3 |
| 11ii | Walker et al. 2010b | 2006 | all 0+ | 183 | 56517 | 107.9 | 92 | 25432.65 | 115.2 | 91 | 31084.35 | 99.7 |
|  |  |  | 0-44 | 30 | 35977.15 | 20.1 | 14 | 11141.5 | 19.5 | 16 | 20470.07 | 20.8 |
|  |  |  | 45-54 | 27 | 11344.54 | 238 | 17 | 9900.99 | 171.7 | 20 | 6172.84 | 324 |
|  |  |  | 55-64 | 33 | 5709.343 | 578 | 17 | 2930.024 | 580.2 | 16 | 2784.061 | 574.7 |
|  |  |  | 65-74 | 55 | 2399.965 | 2291.7 | 23 | 887.1403 | 2592.6 | 22 | 1154.977 | 1904.8 |
|  |  |  | 75-84 | 32 | 840.0053 | 3809.5 | 18 | 431.9965 | 4166.7 | 14 | 400.4004 | 3496.5 |
|  |  |  | 85+ | 6 | 246.0025 | 2439 | 3 | 140.9973 | 2127.7 | 3 | 101.9992 | 2941.2 |
| 12 | Farghaly et al. 2013a | 2007 | 52.5 | 156 | 62583 | 250 | 86 | 32165 | 270 | 70 | 30418 | 230 |
|  | Farghaly et al. 2013b | 2007 | 48.9 | 114 | 44600 | 260 | 63 | 22908 | 280 | 51 | 21692 | 240 |
|  | Farghaly et al. 2013c | 2007 | 54.4 | 42 | 17983 | 230 | 23 | 9257 | 250 | 19 | 8726 | 220 |
| 14i | Osuntokun et al. 1979 | 1975 | all 0+ | 318 | 1223077 | 26 | 229 | 538461.5 | 25 | 89 | 684615.4 | 13 |
|  |  |  | 20-29 | 8 | 800000 | 1 | 5 | 500000 | 1 | 3 | 300000 | 1 |
|  |  |  | 30-39 | 26 | 236363.6 | 11 | 18 | 150000 | 12 | 8 | 100000 | 8 |
|  |  |  | 40-49 | 70 | 104477.6 | 67 | 54 | 60000 | 90 | 16 | 40000 | 40 |
|  |  |  | 50-59 | 80 | 39603.96 | 202 | 55 | 22000 | 250 | 25 | 17857.14 | 140 |
|  |  |  | 60-69 | 87 | 19772.73 | 440 | 60 | 11111.11 | 540 | 27 | 9310.345 | 290 |
|  |  |  | 70-79 | 32 | 6956.522 | 460 | 28 | 3589.744 | 780 | 4 | 3076.923 | 130 |
|  |  |  | 80+ | 10 | 4761.905 | 210 | 5 | 2500 | 200 | 5 | 2380.952 | 210 |
| 15i | Kandil et al. 2006a | 1993 | all 0+ | 39 | 25000 | 180 | 21 | 21000 | 100 | 18 | 21176.47 | 85 |
|  | Kandil et al. 2006b | 1993 |  | 11 | 8464 | 150 | 7 | 7777.778 | 90 | 4 | 7547.17 | 53 |
|  | Kandil et al. 2006c | 1993 |  | 20 | 11228 | 210 | 9 | 9278.351 | 97 | 11 | 9243.697 | 119 |
|  |  |  | 20-39 | 6 | 5774 | 104 |  |  |  |  |  |  |
|  |  |  | 40-59 | 12 | 3073 | 390 |  |  |  |  |  |  |
|  |  |  | 60+ | 19 | 1302 | 1460 |  |  |  |  |  |  |
